# Supplementary figures and images for: Proximity labelling identifies proteins associated with HSV-2 pUL21 at early and late times after infection
Source: PLoS Pathog. 2026 Mar 2;22(3):e1014027. doi: 10.1371/journal.ppat.1014027 (PMC12965700; doi:10.1371/journal.ppat.1014027)

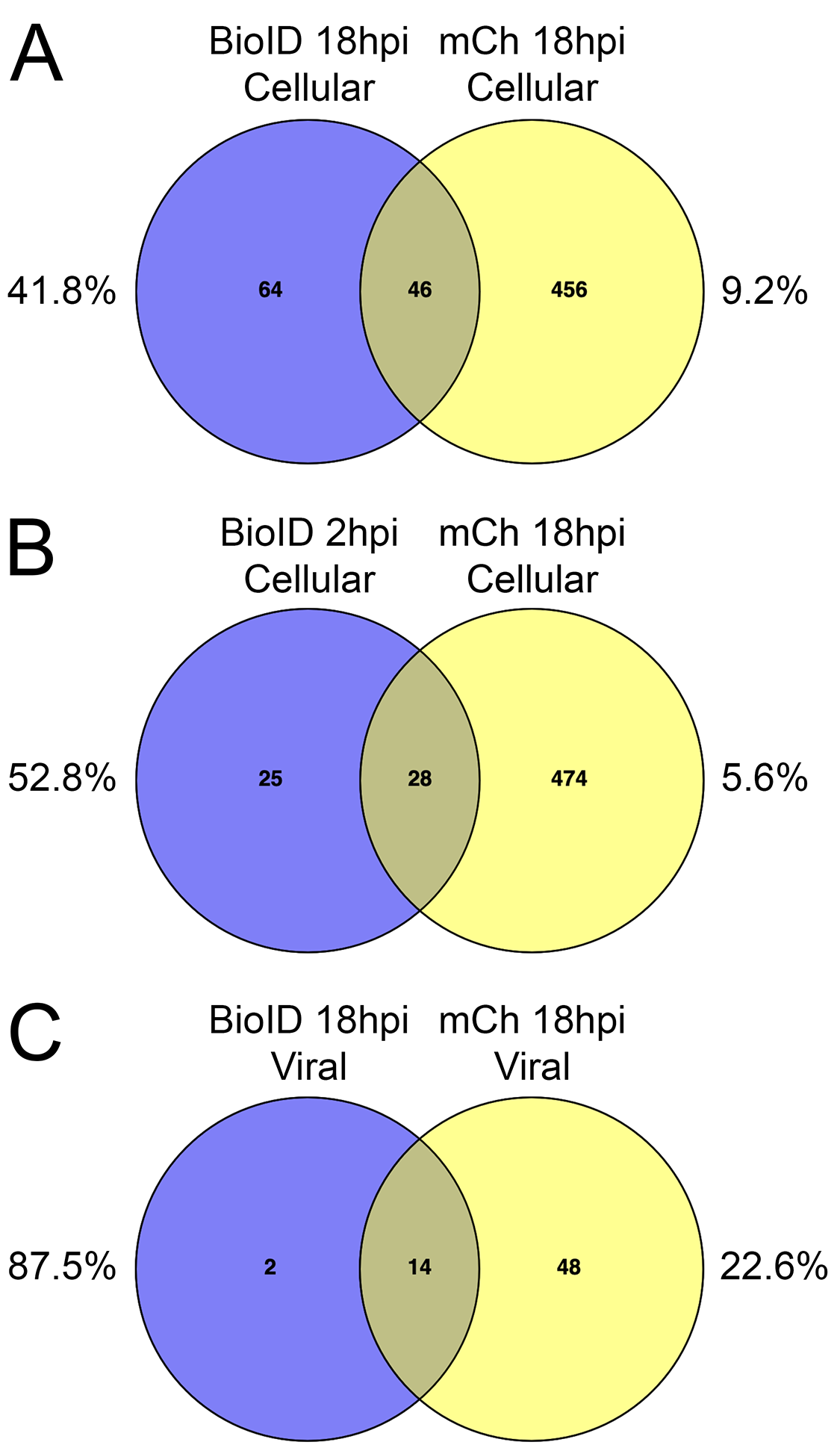

Supplement: S1 Fig — (A) Venn diagram of the number of unique and common cellular proteins in proximity to pUL21mT and interacting with pUL21mCh at 18 hours post-infection (hpi). (B) Venn diagram of the number of unique and common cellular proteins in proximity to tegument-delivered pUL21mT at 2 hpi and interacting with pUL21mCh at 18 hpi. (C) Venn diagram of the number of unique and common viral proteins in proximity to pUL21mT and interacting with pUL21mCh at 18 hpi. Percentages in each panel reflect the proportion of proteins in each dataset that fall within the intersection. (TIF) [file ppat.1014027.s001.tif]

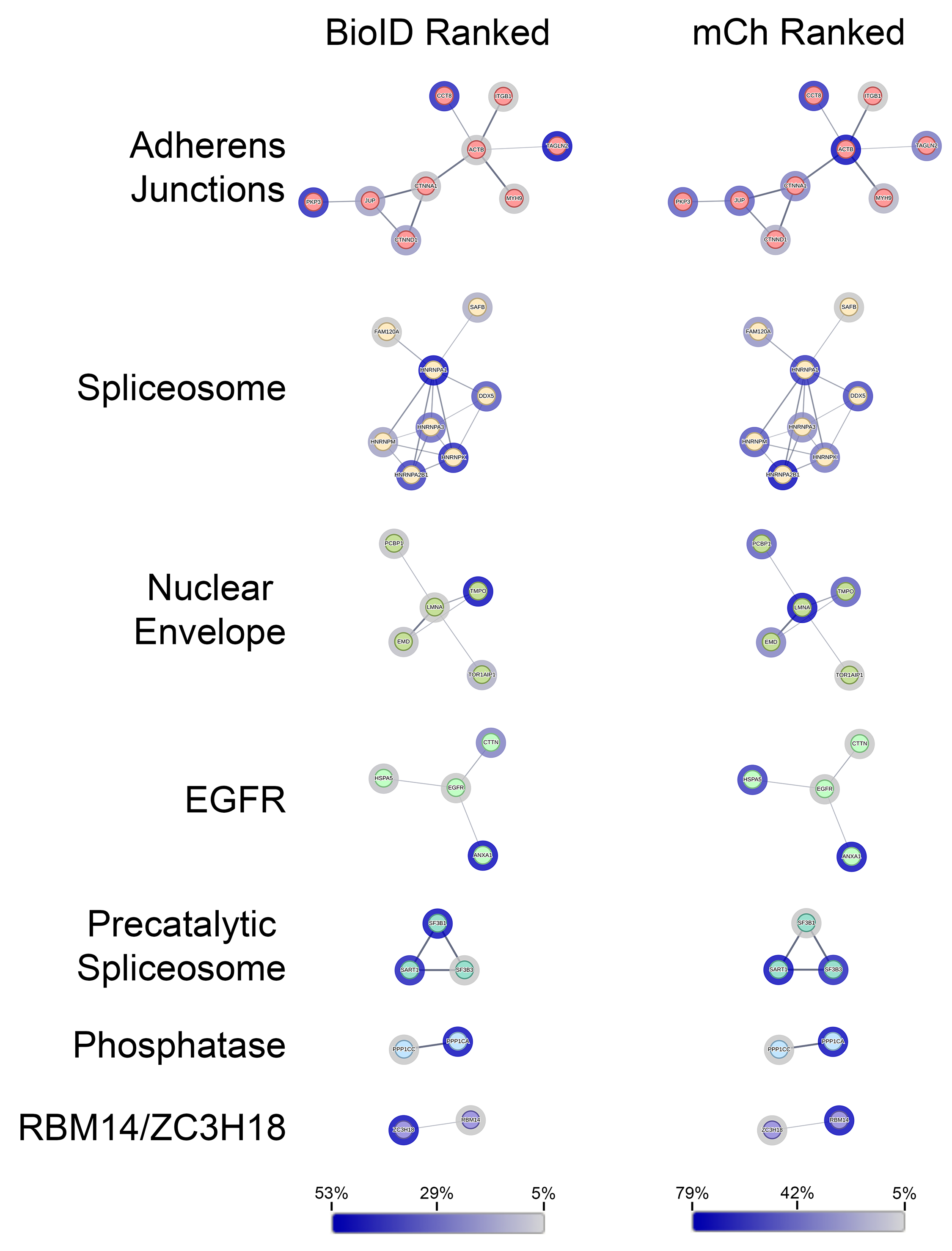

Supplement: S2 Fig — Identified cellular proteins were analyzed, clustered, and colour-coded using the STRING database to visualize known protein-protein interactions. The protein-protein interaction network of each individual cluster is shown. EGFR (epidermal growth factor receptor). Edges connecting the nodes represent reported associations, with edge thickness corresponding to interaction confidence (thicker edges indicate higher confidence). Halos surrounding each node reflect percent coverage, with darker halos indicating higher percent coverage. Only proteins with a normalized or averaged protein coverage of ≥ 5% were included in this analysis, and disconnected nodes are not shown. (TIF) [file ppat.1014027.s002.tif]
