# Supplementary material for: Proximity labelling identifies proteins associated with HSV-2 pUL21 at early and late times after infection
Source: PLoS Pathog. 2026 Mar 2;22(3):e1014027. doi: 10.1371/journal.ppat.1014027 (PMC12965700; doi:10.1371/journal.ppat.1014027)
Supplement: S1 Table — (DOCX) [file ppat.1014027.s003.docx]

Supplementary Table 1. Common Proteins in Proximity to pUL21mT at 18 hpi and 2 hpi

| 18 hpi ^1^Rank | Protein Name | Gene Name | Molecular Weight | ^2^Normalized  Spectral Counts 18 hpi | ^3^Normalized  Percent Coverage ^4^(±SD) 18 hpi | ^2^Normalized  Spectral Counts 2 hpi | ^3^Normalized  Percent Coverage ^4^(±SD) 2 hpi | 2 hpi Rank (^5^∆ rank relative to 18 hpi) |
| --- | --- | --- | --- | --- | --- | --- | --- | --- |
| 1 | Lamina-associated polypeptide 2, isoforms beta/gamma | TMPO | 51 kDa | 71.2 | 53.1 (±1.7) | 40.6 | 57.7 (±1) | 1 (0) |
| 4 | Annexin A1 | ANXA1_HUMAN | 39 kDa | 20.5 | 40 (±1.8) | 5.1 | 12.4 (±4.2) | 28 (-24) |
| 5 | Serine/threonine-protein phosphatase PP1-alpha catalytic subunit | PP1A_HUMAN | 38 kDa | 20.8 | 36.7 (±3.1) | 6.4 | 21.9 (±1.1) | 16 (-11) |
| 6 | T-complex protein 1 subunit theta | TCPQ_HUMAN | 60 kDa | 33.4 | 35.8 (±1.5) | 20.5 | 36.9 (±1.1) | 3 (+3) |
| 7 | Plakophilin-3 | PKP3_HUMAN | 87 kDa | 34.6 | 35.5 (±2.5) | 10.6 | 14.2 (±5.9) | 24 (-17) |
| 8 | Serine/threonine-protein phosphatase PP1-gamma catalytic subunit | PP1G_HUMAN | 37 kDa | 19.7 | 34.9 (±3.4) | 8.5 | 26.3 (±11.5) | 12 (-4) |
| 9 | Heterogeneous nuclear ribonucleoprotein A1 | ROA1_HUMAN | 39 kDa | 54.8 | 30.6 (±2) | 10.2 | 23.1 (±5.2) | 14 (-5) |
| 13 | Inner nuclear membrane protein Man1 | MAN1_HUMAN | 100 kDa | 35.3 | 29.7 (±2.2) | 15.7 | 23.7 (±0) | 13 (0) |
| 14 | Unconventional myosin-VI | MYO6_HUMAN | 150 kDa | 66.1 | 29.3 (±3) | 57 | 33.9 (±0) | 4 (+10) |
| 16 | Tripartite motif-containing protein 29 | TRI29_HUMAN | 66 kDa | 26.7 | 28.6 (±4.4) | 23.3 | 31.5 (±0.8) | 5 (+11) |
| 17 | Pre-mRNA-splicing factor SYF1 | SYF1_HUMAN | 100 kDa | 39.2 | 28.5 (±1.3) | 16.1 | 15.3 (±0) | 22 (-5) |
| 18 | Stromal interaction molecule 1 | STIM1_HUMAN | 77 kDa | 32.7 | 28.4 (±2.9) | 12 | 14.7 (±0) | 23 (-5) |
| 20 | Synaptotagmin-like protein 4 | SYTL4_HUMAN | 76 kDa | 30.3 | 27.6 (±3.4) | 23.2 | 30.3 (±0) | 7 (+13) |
| 21 | Beta/gamma crystallin domain-containing protein 1 | CRBG1_HUMAN | 189 kDa | 63.4 | 27.4 (±0.6) | 20.5 | 14.2 (±7.4) | 25 (-4) |
| 22 | Heterogeneous nuclear ribonucleoprotein K | HNRPK_HUMAN | 51 kDa | 20.6 | 26.7(±2.4) | 6.5 | 16.3 (±3) | 20 (+2) |
| 23 | Src substrate cortactin | SRC8_HUMAN | 62 kDa | 20.4 | 25.1 (±2.2) | 7.6 | 17.5 (±0) | 17 (+6) |
| 24 | Heterogeneous nuclear ribonucleoproteins A2/B1 | ROA2_HUMAN | 37 kDa | 17 | 23.8 (±3.5) | 10.2 | 23.1 (±5.2) | 29 (-5) |
| 26 | Delta(14)-sterol reductase LBR | LBR_HUMAN | 71 kDa | 30.2 | 22.8 (±1.2) | 9.3 | 7.9 (±0.9) | 39 (-13) |
| 29 | Nuclear pore complex protein Nup153 | NU153_HUMAN | 154 kDa | 41.8 | 21.9 (±2.5) | 21.9 | 17 (±0.5) | 19 (+10) |
| 33 | Protein ELYS | ELYS_HUMAN | 253 kDa | 62.2 | 19.3 (±2.5) | 17.7 | 9.1 (±5.8) | 35 (-2) |
| 35 | E3 SUMO-protein ligase RanBP2 | RBP2_HUMAN | 358 kDa | 78.7 | 18.7 (± 1.5) | 55.9 | 15.4 (±3) | 21 (+14) |
| 37 | Emerin | EMD_HUMAN | 29 kDa | 7 | 18 (±1.2) | 5.1 | 22.6 (±0.2) | 15 (+22) |
| 41 | Arf-GAP domain and FG repeat-containing protein 1 | AGFG1_HUMAN | 58 kDa | 17.9 | 17.4 (±0) | 12.7 | 13.3 (±1.4) | 27 (+14) |
| 42 | Lymphoid-specific helicase | HELLS_HUMAN | 97 kDa | 26 | 17.1 (±1.9) | 9.9 | 11.1 (±1.1) | 31 (+11) |
| 45 | Prelamin-A/C | LMNA_HUMAN | 74 kDa | 20.1 | 16.7 (±4.4) | 7.2 | 9.5 (±7.6) | 33 (+12) |
| 46 | Epidermal growth factor receptor | EGFR_HUMAN | 134 kDa | 25.1 | 16.3 (±1.4) | 8.2 | 6.9 (±3.6) | 44 (+2) |
| 50 | Gamma-interferon-inducible protein 16 | IF16_HUMAN | 88 kDa | 49.3 | 16 (±2.2) | 102.3 | 50.3 (±2.5) | 2 (+48) |
| 51 | Ladinin-1 | LAD1_HUMAN | 57 kDa | 9 | 14.8 (±1.1) | 2.3 | 6.1 (±1.2) | 47 (+4) |
| 52 | Catenin delta-1 | CTND1_HUMAN | 108 kDa | 14.1 | 14.2 (±2.4) | 34.7 | 26.8 (±4.7) | 11 (+41) |
| 56 | Misshapen-like kinase 1 | MINK1_HUMAN | 150 kDa | 21.1 | 13.2 (±1) | 5.8 | 5.7 (±0) | 48 (+8) |
| 59 | Junction plakoglobin | PLAK_HUMAN | 82 kDa | 16.2 | 12.7 (±1.5) | 25.4 | 28.6 (±6) | 9 (+50) |
| 69 | Heterogeneous nuclear ribonucleoprotein M | HNRPM_HUMAN | 78 kDa | 9.6 | 10.2 (±1.5) | 4.1 | 5.7 (±7) | 50 (+19) |
| 76 | Microtubule-associated protein 4 | MAP4_HUMAN | 121 kDa | 11.5 | 9.3 (±0.7) | 5.5 | 6.4 (±0.1) | 46 (+30) |
| 92 | Catenin alpha-1 | CTNA1_HUMAN | 100 kDa | 7.5 | 6.5 (±1) | 6.5 | 8.9 (±9.9) | 37 (+55) |
| 101 | Collagen alpha-1(XVII) chain | COHA1_HUMAN | 150 kDa | 12.7 | 5.9 (±1.4) | 18.4 | 10 (±0) | 32 (+69) |

^1^Rank based on normalized percent coverage.

^2^Spectral counts from three biological replicates were normalized to endogenously biotinylated cellular proteins then averaged. Averages of the no-biotin control samples were subtracted from the plus-biotin experimental samples to determine the normalized spectral count.

^3^Average of the percent coverage of three biological replicates of the no-biotin control samples was subtracted from the average of the percent coverage of the plus-biotin experimental samples to determine the normalized percent coverage.

^4^Standard deviation (SD) of the three plus-biotin biological replicates.

^5^Change in protein rank based on percent coverage in 2 hpi samples relative to 18 hpi samples
