# Supplementary material for: Proximity labelling identifies proteins associated with HSV-2 pUL21 at early and late times after infection
Source: PLoS Pathog. 2026 Mar 2;22(3):e1014027. doi: 10.1371/journal.ppat.1014027 (PMC12965700; doi:10.1371/journal.ppat.1014027)
Supplement: S2 Table — (DOCX) [file ppat.1014027.s004.docx]

Supplementary Table 2. Top 10 Unique Proteins in Proximity to pUL21mT at 18 hpi

| ^1^Rank | Protein Name | Gene Name | Molecular Weight | ^2^Normalized Spectral Counts | ^3^Normalized  Percent Coverage ^4^(±SD) |
| --- | --- | --- | --- | --- | --- |
| 1 | Cytoplasmic dynein 1 light intermediate chain 2 | DC1L2_HUMAN | 54 kDa | 27.9 | 44.5 (±6) |
| 2 | Transgelin-2 | TAGL2_HUMAN | 22 kDa | 11.6 | 41 (±5.5) |
| 3 | Protein LYRIC | LYRIC_HUMAN | 64 kDa | 22.8 | 29.8 (±5.7) |
| 4 | Signal recognition particle receptor subunit alpha | SRPRA_HUMAN | 70 kDa | 27 | 29.8 (±3.5) |
| 5 | U4/U6 small nuclear ribonucleoprotein Prp3 | PRPF3_HUMAN | 78 kDa | 27.1 | 28.7(±2.1) |
| 6 | Transcription factor jun-B | JUNB_HUMAN | 36 kDa | 10 | 28 (±0) |
| 7 | Proliferation marker protein Ki-67 | KI67_HUMAN | 359 kDa | 115.6 | 23.8 (±3.6) |
| 8 | SUN domain-containing protein 1 | SUN1_HUMAN | 90 kDa | 23.2 | 22.5 (±3.4) |
| 9 | Myristoylated alanine-rich C-kinase substrate | MARCS_HUMAN | 32 kDa | 7.4 | 22.3(±2.6) |
| 10 | Torsin-1A-interacting protein 1 | TOIP1_HUMAN | 66 kDa | 17.1 | 21.6 (±2.3) |

^1^Rank based on normalized percent coverage.

^2^Spectral counts from three biological replicates were normalized to endogenously biotinylated cellular proteins then averaged. Averages of the no-biotin control samples were subtracted from the plus-biotin experimental samples to determine the normalized spectral count.

^3^Average of the percent coverage of three biological replicates of the no-biotin control samples was subtracted from the average of the percent coverage of the plus-biotin experimental samples to determine the normalized percent coverage.

^4^ Standard deviation (SD) of the three plus-biotin biological replicates.
