# Supplementary material for: Proximity labelling identifies proteins associated with HSV-2 pUL21 at early and late times after infection
Source: PLoS Pathog. 2026 Mar 2;22(3):e1014027. doi: 10.1371/journal.ppat.1014027 (PMC12965700; doi:10.1371/journal.ppat.1014027)
Supplement: S3 Table — (DOCX) [file ppat.1014027.s005.docx]

Supplementary Table 3. Top 10 Unique Proteins in Proximity to pUL21mT at 2 hpi

| ^1^Rank | Protein Name | Gene Name | Molecular Weight | ^2^Normalized  Spectral Counts | ^3^Normalized  Percent Coverage ^4^(±SD) |
| --- | --- | --- | --- | --- | --- |
| 1 | Thioredoxin | THIO_HUMAN | 12 kDa | 7.2 | 31.4 (±0.5) |
| 2 | Erbin | ERBIN_HUMAN | 158 kDa | 37.8 | 29.3 (±7.8) |
| 3 | Pleckstrin homology domain-containing family A member 5 | PKHA5_HUMAN | 127 kDa | 33.8 | 28.1 (±0.7) |
| 4 | Myosin light polypeptide 6 | MYL6_HUMAN | 17 kDa | 2.4 | 17.2 (±0.3) |
| 5 | Sickle tail protein homolog | SKT_HUMAN | 214 kDa | 26.9 | 14 (±0) |
| 6 | Transmembrane protein 263 | TM263_HUMAN | 12 kDa | 2.4 | 11.8 (±1.1) |
| 7 | Plakophilin-1 | PKP1_HUMAN | 83 kDa | 5.5 | 9.5 (±0) |
| 8 | Rho GTPase-activating protein 21 | RHG21_HUMAN | 217 kDa | 15.1 | 8.9 (±1.9) |
| 9 | Protein FAM83H | FA83H_HUMAN | 127 kDa | 7.9 | 8.6 (±0) |
| 10 | Tubulin beta-4B chain | TBB4B_HUMAN | 50 kDa | 2.8 | 7.5 (±0) |

^1^Rank based on normalized percent coverage.

^2^Spectral counts from three biological replicates were normalized to endogenously biotinylated cellular proteins then averaged. Averages of the no-biotin control samples were subtracted from the plus-biotin experimental samples to determine the normalized spectral count.

^3^Average of the percent coverage of three biological replicates of the no-biotin control samples was subtracted from the average of the percent coverage of the plus-biotin experimental samples to determine the normalized percent coverage.

^4^ Standard deviation (SD) of the three plus-biotin biological replicates.
