# Supplementary material for: Proximity labelling identifies proteins associated with HSV-2 pUL21 at early and late times after infection
Source: PLoS Pathog. 2026 Mar 2;22(3):e1014027. doi: 10.1371/journal.ppat.1014027 (PMC12965700; doi:10.1371/journal.ppat.1014027)
Supplement: S4 Table — (DOCX) [file ppat.1014027.s006.docx]

Supplementary Table 4. Common Cellular Proteins Identified by BioID at 18 hpi and Affinity-Purified with pUL21mCh at 18 hpi

| ^1^Rank | Gene Name |
| --- | --- |
| 1 | TMPO |
| 2 | TAGL2_HUMAN |
| 3 | ANXA1_HUMAN |
| 4 | PP1A_HUMAN |
| 5 | TCPQ_HUMAN |
| 6 | PKP3_HUMAN |
| 7 | PP1G_HUMAN |
| 8 | ROA1_HUMAN |
| 9 | LYRIC_HUMAN |
| 10 | SRPRA_HUMAN |
| 11 | TRI29_HUMAN |
| 12 | HNRPK_HUMAN |
| 13 | SRC8_HUMAN |
| 14 | ROA2_HUMAN |
| 15 | MARCS_HUMAN |
| 16 | TOIP1_HUMAN |
| 17 | EMD_HUMAN |
| 18 | ROA3_HUMAN |
| 19 | PCBP1_HUMAN |
| 20 | SF3B1_HUMAN |
| 21 | BIP_HUMAN |
| 22 | DDX3X_HUMAN |
| 23 | LMNA_HUMAN |
| 24 | EGFR_HUMAN |
| 25 | SNUT1_HUMAN |
| 26 | LAD1_HUMAN |
| 27 | CTND1_HUMAN |
| 28 | DDX18_HUMAN |
| 29 | PLAK_HUMAN |
| 30 | ZCH18_HUMAN |
| 31 | PAI2_HUMAN |
| 32 | TR150_HUMAN |
| 33 | HNRPM_HUMAN |
| 34 | RBM14_HUMAN |
| 35 | SAFB1_HUMAN |
| 36 | NAT10_HUMAN |
| 37 | FAS_HUMAN |
| 38 | PEPL_HUMAN |
| 39 | CTNA1_HUMAN |
| 40 | NUCL_HUMAN |
| 41 | SF3B3_HUMAN |
| 42 | COHA1_HUMAN |
| 43 | MYH9_HUMAN |
| 44 | ADNP_HUMAN |
| 45 | ITB1_HUMAN |
| 46 | F120A_HUMAN |

^1^Proteins ranked in order of normalized percent coverage obtained in 18 hpi BioID experiment.
