# Supplementary material for: Proximity labelling identifies proteins associated with HSV-2 pUL21 at early and late times after infection
Source: PLoS Pathog. 2026 Mar 2;22(3):e1014027. doi: 10.1371/journal.ppat.1014027 (PMC12965700; doi:10.1371/journal.ppat.1014027)
Supplement: S5 Table — (DOCX) [file ppat.1014027.s007.docx]

Supplementary Table 5. Common Viral Proteins Identified by BioID at 18 hpi and Affinity-Purified with pUL21mCh at 18 hpi

| ^1^Rank | Protein Name | Gene Name |
| --- | --- | --- |
| 1 | pUL21 | TEG4_HHV2H |
| 2 | pUL51 | TEG7_HHV2H |
| 3 | pUL34 | NEC2_HHV2H |
| 4 | pUL38 (triplex capsid protein 1) | TRX1_HHV2G |
| 5 | pUL2 (uracil DNA glycosylase) | UNG_HHV2H |
| 6 | pUL37 | ITP_HHV2H |
| 7 | pUL47 | TEG5_HHV2H |
| 8 | pUL42 | PAP_HHV2H |
| 9 | pUL27 (gB) | GB_HHV23 |
| 10 | pUL31 | NEC1_HHV2H |
| 11 | pUL39 (ribonucleotide reductase large subunit) | RIR1_HHV23 |
| 12 | pUL30 (DNA polymerase catalytic subunit) | DPOL_HHV21 |
| 13 | pUL49 (VP22) | VP22_HHV2H |
| 14 | pUL36 (large tegument protein) | LTP_HHV2H |

^1^Proteins ranked in order of normalized percent coverage obtained in 18 hpi BioID experiment.
