# Supplementary material for: Proximity labelling identifies proteins associated with HSV-2 pUL21 at early and late times after infection
Source: PLoS Pathog. 2026 Mar 2;22(3):e1014027. doi: 10.1371/journal.ppat.1014027 (PMC12965700; doi:10.1371/journal.ppat.1014027)
Supplement: S6 Table — (DOCX) [file ppat.1014027.s008.docx]

Supplementary Table 6. Common Cellular Proteins Identified by BioID at 2 hpi and Affinity-Purified with pUL21mCh

| ^1^Rank | Gene Name |
| --- | --- |
| 1 | TMPO |
| 2 | TCPQ_HUMAN |
| 3 | TRI29_HUMAN |
| 4 | PLAK_HUMAN |
| 5 | CTND1_HUMAN |
| 6 | PP1G_HUMAN |
| 7 | ROA1_HUMAN |
| 8 | EMD_HUMAN |
| 9 | PP1A_HUMAN |
| 10 | SRC8_HUMAN |
| 11 | MYL6_HUMAN |
| 12 | HNRPK_HUMAN |
| 13 | PKP3_HUMAN |
| 14 | ANXA1_HUMAN |
| 15 | ROA2_HUMAN |
| 16 | COHA1_HUMAN |
| 17 | LMNA_HUMAN |
| 18 | CTNA1_HUMAN |
| 19 | FA83H_HUMAN |
| 20 | TBB4B_HUMAN |
| 21 | CAV1_HUMAN |
| 22 | EGFR_HUMAN |
| 23 | ANXA2_HUMAN |
| 24 | LAD1_HUMAN |
| 25 | EF1A1_HUMAN |
| 26 | HNRPM_HUMAN |
| 27 | SPB5_HUMAN |
| 28 | CAZA1_HUMAN |

^1^Proteins ranked in order of normalized percent coverage obtained in 2 hpi BioID experiment.
